# The impact of age in acute type A aortic dissection: a retrospective study

**DOI:** 10.1186/s13019-022-01785-y

**Published:** 2022-03-19

**Authors:** Jun-Xia Wang, Yun-Xing Xue, Xi-Yu Zhu, Ho-Shun Chong, Zhong Chen, Qing Zhou, Jason-Zhensheng Qu, Dong-Jin Wang

**Affiliations:** 1 Department of Cardiothoracic Surgery, Affiliated Drum Tower Hospital, Medical School of Nanjing University, 321 Zhongshan Road, Nanjing, 210000 Jiangsu China; 2 Institute of Cardiothoracic Vascular Disease, Nanjing University, Nanjing, China; 3 Department of Anesthesia, Critical Care and Pain Medicine, Massachusetts General Hospital, Boston, MA USA

**Keywords:** Aortic dissection, Age, Surgical therapy

## Abstract

**Background:**

Acute type A aortic dissection (aTAAD) is a lethal disease and age is an important risk factor for outcomes. This retrospective study was to analyze the impact of age stratification in aTAAD, and to provide clues for surgeons when they make choices of therapy strategies.

**Methods:**

From January 2011 to December 2019, 1092 aTAAD patients from Nanjing Drum Tower Hospital received surgical therapy. Patients were divided into 7 groups according to every ten-year interval (20–80 s). The differences between the groups were analyzed in terms of the baseline preoperative conditions, surgical methods and postoperative outcomes of patients of different age groups. During a median follow-up term of 17 months, the survival rates were compared among 7 groups through Kaplan–Meier analysis.

**Results:**

The median age was 52.0 years old in whole cohort. The multiple comorbidities were more common in old age groups (60 s, 70 s, 80 s), while the 20 s group patients had the highest proportion of Marfan syndrome (28.1%). Preoperative hypotension was highest in 80 s (16.7%, *P* = 0.038). Young age groups (20–60 s) had a higher rate of root replacement and total arch replacement, which led to a longer duration of operation and hypothermic circulation arrest. The overall mortality was 14.1%, the tendency of mortality was increased with age except 20 s group (33.3% in 80 s, *P* = 0.016). The postoperative morbidity of gastrointestinal bleeding and bowel ischemia were 16.7% and 11.1% in 80 s group.

**Conclusions:**

Age is a major impact factor for aTAAD surgery. Old patients presented more comorbidities before surgery, the mortality and complications rate were significantly higher even with less invasive and conservative surgical therapy. But the favorable long-term survival indicated that the simple or less extensive arch repair is the preferred surgery for patients over 70 years old.

**Supplementary Information:**

The online version contains supplementary material available at 10.1186/s13019-022-01785-y.

## Background

Age has shown to be a strong independent impact factor of outcomes in acute type A aortic dissection (aTAAD), while treatment strategies and surgical methods differentiate according to age stratification. Old age has long been thought to increase the risk of surgery, which likely explains the higher rate of medical therapy especially in patients older than 70 years old [1]. As the aging of population increases, surgical techniques and perioperative management continue to improve, advanced age is no longer a major deterrent of surgical repair of aTAAD albeit conservative therapy is chosen by patients and physicians [2, 3]. Studies have shown that the average age of patients with aTAAD is 55 years old in China, a decade younger than that in the western countries [4, 5]. So an extensive one-stage surgery, total arch replacement and frozen elephant trunk, has become the preferred surgical strategy in China to avoid reintervention [6, 7]. There are also reports on one-stage total aortic arch replacement in other counties.

However, as the number of patients with aTAAD increases in age groups in China, especially in the groups of 60–80 years of age, the long-term survival of this extensive surgery is largely unknown. Should age be factored in the consideration before such an extensive surgery be planed? The age-stratified clinical characteristics, treatment strategies, and outcomes in Chinese patients are not yet known. The aim of this retrospective study is to investigate the clinical characteristics, treatment strategy and surgical outcomes of aTAAD in different age stratification groups in our center.

## Methods

### Patients

Between January 2011 and December 2019, a total of 1174 patients with aTAAD were admitted to Nanjing Drum Tower Hospital (NDTH). 1092 patients who underwent open surgical repair were divided into seven groups according to every ten-year interval (20–80 s) and 82 patients who did not undergo surgical therapy were excluded. Diagnosis of aTAAD was confirmed by computed tomographic angiography (CTA) scanning within two weeks after the onset of symptoms.

All clinical data were collected prospectively by admission and during the in-hospital stay. We retrieved the data retrospectively by review of hospital records. The study was conducted in accordance with the Declaration of Helsinki (as revised in 2013). The current study was approved by the institutional review board of Nanjing Drum Tower Hospital (2020-185-01).

### Treatment

Patients diagnosed with aTAAD were transferred to cardiac surgery intensive care unit and optimal medical therapy was initiated. Those with the signs of severe low blood pressure and tamponade will be taken in the operating room direct from emergency. Open surgery was recommended for all patients, but for patients with advanced age, dissection associated organ malperfusion or family refusal, medical therapy was the treatment of choice. The patients received open surgery underwent general anesthesia via a standard median sternotomy after signing the informed consents. Cardiopulmonary bypass (CPB) was initiated with femoral artery or axillary artery arterial cannulation and right atrium or superior/inferior venous cannulation. Deep or mild hypothermic circulatory arrest (HCA) was used in all patients. Selective antegrade or retrograde cerebral perfusion was applied for brain protection during the period of HCA at operating surgeon’s choice. The distal aortic arch surgical strategy included partial arch replacement, total arch replacement with or without frozen elephant trunk (Microport Corp.Ltd, Shanghai, China) and arch stent (Yuhengjia Sci Tech Corp.Ltd, Beijing, China) based on the pathological involvement of the aortic arch [7–9]. After finishing the distal repair, the re-warming stage begun as the proximal part of aorta or root was being reconstructed and the patients were weaned off CPB. Bentall procedure or root reinforcement reconstruction was applied based on the anatomic indications [10, 11]. The patients were transferred to the floor after recovering in cardiac surgery ICU, and discharged from the hospital per institution protocol.

### Statistical analysis

Statistical analysis was performed with SPSS 26.0 (IBM Corp. Released 2019. IBM SPSS Statistics for Macintosh, Version 26.0. Armonk, NY: IBM Corp.). Descriptive statistics were used to describe patient characteristics throughout the study. Means and standard deviations were presented for normally distributed continuous variables whereas median and the interquartile ranges were computed to describe non-normally distributed continuous data. Categorical data are presented as frequency distributions and simple percentages. Between group differences were analyzed using a Student’s t-test, Kruskal–Wallis H test or Mann–Whitney U-test for continuous variables and a Chi-square or Fisher’s exact test for categorical variables. The survival curve was draw using Kaplan–Meier method and compared using the log-rank test. The median follow-up time was calculated with reverse Kaplan–Meier method. Statistical significance was considered when *P* < 0.05.

## Results

### Demographics and Preoperative characteristics

**Fig. 1:**
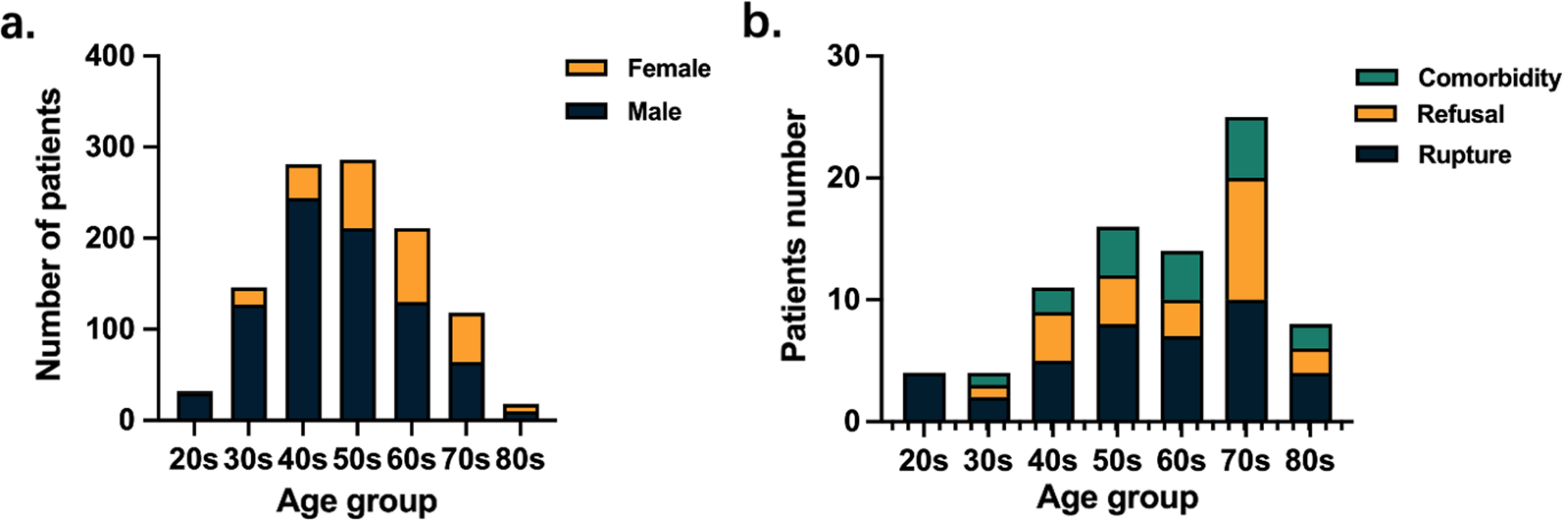
**a** Number and sex ratio of patients in different age groups of our center; **b** The reason for non-surgery after admission. The main reason was a rupture

Eighty-two of the 1174 aTAAD patients chose medical management that was chosen by 30.8% of patients in 80 years group due to rupture of the dissection (Fig. 1a, b). The patients aged 40–60 years constituted the largest proportion of patients (71.2%) and the youngest 20 s (2.9%) and oldest 80 s (1.6%) groups accounted for minimum percentage of patients. There was higher proportion of female patients as age increases.

Further analysis showed that the 20 s group patients had the highest proportion of connective tissue diseases (Marfan’s syndrome) (28.1%). History of hypertension was present in 63% patients ages between 40 and 70 years old and hypotension on admission was highest in 80 s group (16.7%, *P* = 0.038) (Table 1). The average BMI was 25.6 with the highest 33.1 in 30 s group and lowest 19.5 in 80 s group. There is significantly increased history of stroke (9.6% vs 1.9%, OR 5.5, 95% CI 2.6–11.5), coronary artery disease (CAD) (5.9% vs 2.0%, OR 3.1, 95% CI 1.3–7.2) in patients over 70 years old.

**Table 1:** Baseline demographics and characteristics

	Total	20 s	30 s	40 s	50 s	60 s	70 s	80 s	P value
Number	1092	32	146	281	286	211	118	18	
Age	52 (62–44)	27 (28–25)	35.5(38–32)	45 (47–43)	54 (56–52)	64 (66–62)	74 (76–71)	82.5(84.25–80)	< 0.001
Male	816 (74.7%)	30 (93.8%)	127 (87.0%)	244 (86.8%)	211 (73.8%)	130 (61.6%)	64 (54.2%)	10 (55.6%)	< 0.001
BMI (kg/m^2^)	25.6 ± 4.6	25.8 ± 5.4	27.4 ± 5.7	26.4 ± 4.7	25.4 ± 4.1	24.7 ± 4.0	24.1 ± 3.9	23.4 ± 3.9	< 0.001
Hypertension	807 (73.9%)	12 (37.5%)	95 (65.1%)	215 (76.5%)	219 (76.6%)	162 (76.8%)	92 (78.0%)	12 (66.7%)	< 0.001
Marfan	26 (2.4%)	9 (28.1%)	4 (2.7%)	10 (3.6%)	2 (0.7%)	1 (0.5%)	0 (0%)	0 (0%)	< 0.001
Diabetes	40 (3.7%)	0 (0%)	3 (2.1%)	8 (2.8%)	9 (3.1%)	11 (5.2%)	7 (5.9%)	2 (11.1%)	0.181
Smoke	250 (22.9%)	10 (31.3%)	34 (23.3%)	77 (27.4%)	71 (24.8%)	40 (19.0%)	14 (11.9%)	4 (22.2%)	0.019
Alcohol	167 (15.3%)	3 (9.4%)	28 (19.2%)	44 (15.7%)	54 (18.9%)	25 (11.8%)	11 (9.3%)	2 (11.1%)	0.098
End stage kidney disease	23 (2.1%)	0 (0.0%)	4 (2.7%)	5 (1.8%)	11(3.8%)	0 (0.0%)	3 (2.5%)	0 (0.0%)	0.072
Stroke history	31 (2.8%)	0 (0%)	1 (0.7%)	5 (1.8%)	7 (2.4%)	5 (2.4%)	10 (8.5%)	3 (16.7%)	0.001
CAD history	27 (2.5%)	0 (0%)	2 (1.4%)	3 (1.1%)	5 (1.7%)	9 (4.3%)	6 (5.1%)	2 (11.1%)	0.020
COPD history	11 (1.0%)	0 (0%)	1 (0.7%)	2 (0.7%)	3 (0.3%)	3 (1.4%)	2 (3.4%)	0 (0%)	0.919
AF history	10 (0.9%)	0 (0%)	1 (0.7%)	1 (0.4%)	2 (0.7%)	3 (1.4%)	2 (1.7%)	1 (5.6%)	0.273
Pain	1007 (92.3%)	28 (87.5%)	134 (91.8%)	262 (93.2%)	264 (92.3%)	194 (91.9%)	109 (92.4%)	17 (94.4%)	0.936
Chest	942 (86.3%)	28 (87.5%)	125 (85.6%)	244 (86.8%)	251 (87.8%)	179 (84.8%)	102 (86.4%)	15 (83.3%)	0.965
Back	433 (39.7%)	9 (28.1%)	59 (40.4%)	110 (39.1%)	122 (42.7%)	78 (37.0%)	50 (42.4%)	5 (27.8%)	0.565
Abdominal	59 (5.4%)	0 (0%)	10 (6.8%)	19 (6.8%)	12 (4.2%)	10 (4.7%)	5 (4.2%)	3 (16.7%)	0.197
Leg	32 (2.9%)	2 (6.3%)	5 (3.4%)	9 (3.2%)	8 (2.8%)	6 (2.8%)	2 (1.7%)	0 (0%)	0.848
Malperfusion									
Cerebral	108 (9.9%)	0 (0%)	9 (6.2%)	26 (9.3%)	32 (11.2%)	21 (10%)	19 (16.1%)	1 (5.6%)	0.062
Limb	165 (15.1%)	7 (21.9%)	20 (13.7%)	49 (17.4%)	46 (16.1%)	30 (14.2%)	12 (10.2%)	1 (5.6%)	0.397
Bowel	47 (4.3%)	0 (0%)	4 (2.7%)	13 (4.6%)	13 (4.5%)	10 (4.7%)	7 (5.9%)	0 (0%)	0.666
Myocardial	52 (4.8%)	2 (6.3%)	11 (7.5%)	10 (3.6%)	14 (4.9%)	10 (4.7%)	5 (4.2%)	0 (0%)	0.646
Hypotension	64 (5.9%)	1 (3.1%)	4 (2.7%)	11 (3.9%)	20 (7.0%)	19 (9.0%)	6 (5.1%)	3 (16.7%)	0.040
Pericardial tamponade	131 (12.0%)	4 (12.5%)	5 (3.4%)	26 (9.3%)	39 (13.6%)	34 (16.1%)	22 (18.6%)	1 (5.6%)	0.001
Coronary artery involvement	215 (19.7%)	11 (34.4%)	28 (19.2%)	53 (18.9%)	62 (21.7%)	39 (18.5%)	21 (17.8%)	1 (5.6%)	0.278

*BMI* body mass index, *CAD* coronary artery disease, *COPD* chronic obstructive pulmonary disease

Pain was the main presenting symptom, while chest pain presented as similar among groups. Preoperative malperfusion were present in 34% of patients with no significant difference among age groups.

### Operative characteristics

The duration of surgery, CPB, X-clamp and HCA decreased with advanced age starting from age group of 50 years old. Cannulating both femoral and axillary artery were preferred arterial cannulation approach compared to single femoral or axillary artery. Bentall procedure accounted for a large part of root methods in young age group (43.8% in 20 s) and total arch replacement with FET had a higher rate in age group of 50 s (45.8%), 60 s (41.7%) than the 70 s (26.3%) and 80 s (16.7%) (*P* < 0.001) (Table 2).

**Table 2:** Operative data

	Total	20 s	30 s	40 s	50 s	60 s	70 s	80 s	P value
Number	1092	32	146	281	286	211	118	18	
Hour from onset to admission	10 (18–6)	10 (20–6)	10 (18–7)	10 (18–7)	9 (16–6)	10 (20–6)	10 (18–6)	9 (12–5)	0.374
Hours from admission to surgery	6 (12–3)	9 (12–3)	6 (13–3)	6 (14–3)	5 (12–3)	5 (12–2)	5 (10–3)	6 (12–3)	0.049
OP duration	8.0 ± 2.1	8.2 ± 2.1	8.2 ± 2.1	8.3 ± 2.2	7.8 ± 2.3	7.7 ± 1.9	7.7 ± 1.9	7.1 ± 1.6	< 0.001
Cannulation									
Ascending	20 (1.8%)	2 (6.3%)	3 (2.1%)	5 (1.8%)	3 (1.0%)	5 (2.4%)	2 (1.7%)	0 (0%)	0.521
Femoral	231 (21.2%)	7 (21.9%)	18 (12.3%)	36 (12.8%)	68 (23.8%)	61 (28.9%)	33 (28.0%)	8 (44.4%)	0.000
Axillary	233 (21.3%)	3 (9.4%)	25 (17.1%)	64 (22.8%)	56 (19.6%)	50 (23.7%)	30 (25.4%)	5 (27.8%)	0.281
Femoral + axillary	608 (55.7%)	20 (62.5%)	100 (68.5%)	176 (62.6%)	159 (55.6%)	95 (45.0%)	53 (44.9%)	5 (27.8%)	0.000
HCA	30.3 ± 11.1	29.2 ± 15.6	32.4 ± 12.6	30.7 ± 10.8	29.7 ± 10.8	30.6 ± 11.1	28.7 ± 8.5	24.8 ± 8.7	0.021
CPB	240.0 ± 76.1	238.8 ± 64.4	253.6 ± 74.2	245.9 ± 81.4	237.7 ± 76.7	234.7 ± 75.9	228.3 ± 66.9	219.3 ± 61.1	0.012
X-clamp	166.8 ± 61.1	168.5 ± 47.4	179.8 ± 59.3	169.8 ± 72.3	164.1 ± 57.6	163.1 ± 57.8	157.8 ± 49.1	159.7 ± 59.8	0.049
Cerebral perfusion									< 0.001
No perfusion	140 (12.8%)	7 (21.9%)	10 (6.8%)	23 (8.2%)	43 (15.0%)	39 (18.5%)	14 (11.9%)	4 (22.2%)	
ACP	886 (81.1%)	25 (78.1%)	130 (89.0%)	250 (89.0%)	226 (79.0%)	153 (72.5%)	91 (77.1%)	11 (61.1%)	
RCP	66 (6.0%)	0 (0%)	6 (4.1%)	8 (2.8%)	17 (5.9%)	19 (9.0%)	13 (11.0%)	3 (16.7%)	
Root procedure									< 0.001
No	17 (1.6%)	2 (6.3%)	2 (1.4%)	5 (1.8%)	3 (1.0%)	3 (1.4%)	2 (1.7%)	0 (0%)	
Root reconstruction	828 (75.8%)	13 (40.6%)	99 (67.8%)	213 (75.8%)	214 (74.8%)	174 (82.5%)	101 (85.6%)	14 (77.8%)	
Bentall	228 (20.9%)	14 (43.8%)	38 (26.0%)	60 (21.4%)	68 (23.8%)	30 (14.2%)	14 (11.9%)	4(22.2%)	
VSRR	19 (1.7%)	3 (9.4%)	7 (4.8%)	3 (1.1%)	1 (0.3%)	4 (1.9%)	1 (0.8%)	0 (0%)	
Arch procedure									< 0.001
Sub-arch	211 (19.9%)	7 (21.9%)	18 (12.3%)	40 (14.2%)	49 (17.1%)	47 (22.3%)	45 (38.1%)	7 (38.9%)	
Total arch + FET	515 (47.1%)	19 (59.4%)	88 (60.3%)	156 (55.5%)	131 (45.8%)	88 (41.7%)	31 (26.3%)	3 (16.7%)	
Arch stent	361 (33.0%)	6 (18.8%)	40 (27.4%)	85 (30.2%)	106 (37.1%)	74 (35.1%)	41 (34.7%)	8 (44.4%)	

*OP* operation, *HCA* hypothermic circulatory arrest, *CPB* cardiopulmonary bypass, *ACP* antegrade cerebral perfusion, *RCP* retrograde cerebral perfusion, *VSRR* valve sparing root reconstruction, *FET* frozen elephant trunk technique

### Immediate postoperative outcomes

**Fig. 2:**
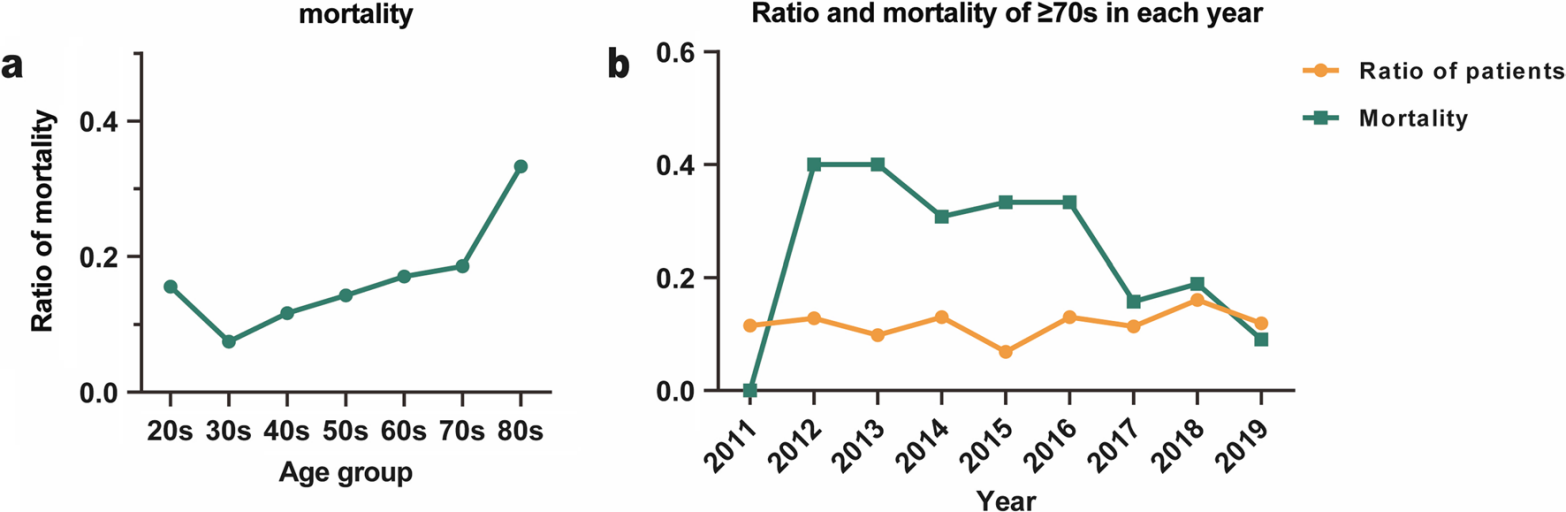
**a** Ratio of mortality in different age groups. **b** Ratio and mortality of ≥ 70 years old patients each year, and the overall mortality fluctuation from 2011 to 2019

One hundred and fifty-four patients (14.1%) died within 30 days after surgery, 93 (60.4%) from circulatory failure, 21 (13.6%) neurological complications, 15 (9.7%) aortic rupture, 13 (8.4%) respiratory failure or other reasons, 12 (7.8%) gastrointestinal bleeding or ischemia (Table 3). Group 80 s had significantly higher mortality than group 70 s (33.3% vs 18.6%, *P* = 0.016) (Fig. 2a). A decreasing trend of mortality rate in 70 s and 80 s group was shown with year (Fig. 2b). Age was related to postoperative complications. The stroke rates (died and not died) were 8.5%, 5.1% and 5.6% in 60 s, 70 s and 80 s group respectively. Among patients succumbed in the 80-year group, there were significant high rate of GI bleeding and ischemia (16.7% and 11.1%, respectively) compared with other groups of 50 s, 60 s and 70 s (1.4%, 0%, 0.8%, respectively). The 70 s group had higher duration of ICU stay when compared with other groups (20 s group: *P* = 0.048, 30 s group: *P* = 0.047, 50 s group: *P* = 0.011); however, the duration of hospital stay showed no significant difference in all age groups. There was no significant difference in mechanical ventilation, reintubation, tracheotomy, neurological complications, renal complications and re-exploration. The 20 s group showed lowest postoperative neurological and gastrointestinal complications as they presented at admission (Table 3).

**Table 3:** Postoperative data

	Total	20 s	30 s	40 s	50 s	60 s	70 s	80 s	P value
Number	1092	32	146	281	286	211	118	18	
30 day-mortality	154 (14.1%)	5 (15.6%)	11 (7.5%)	33 (11.7%)	41 (14.3%)	36 (17.1%)	22 (18.6%)	6 (33.3%)	0.016
Mechanical ventilation	56.2 ± 79.1	71.6 ± 130.2	61.2 ± 90.4	55.2 ± 69.6	57.5 ± 93.8	52.9 ± 63.3	49.3 ± 51.0	67.6 ± 80.4	0.808
Reintubation	70 (6.4%)	4 (12.5%)	6 (4.1%)	14 (5.0%)	23 (8.0%)	12 (5.7%)	11 (9.3%)	0 (0%)	0.199
Tracheotomy	44 (4.0%)	2 (6.3%)	4 (2.7%)	11 (3.9%)	10 (3.5%)	7 (3.3%)	10 (8.5%)	0 (0%)	0.216
ICH	9 (0.8%)	0 (0%)	1 (0.7%)	1 (0.4%)	5 (1.7%)	1 (0.5%)	1 (0.8%)	0 (0%)	0.609
Stroke	59 (5.4%)	0 (0%)	3 (2.1%)	14 (5.0%)	17 (5.9%)	18 (8.5%)	6 (5.1%)	1 (5.6%)	0.155
Paraplegia	23 (2.1%)	0 (0%)	4 (2.7%)	9 (3.2%)	7 (2.4%)	2 (0.9%)	1 (0.8%)	0 (0%)	0.489
GI bleeding	13 (1.2%)	0 (0%)	1 (0.7%)	4 (1.4%)	4 (1.4%)	0 (0%)	1 (0.8%)	3 (16.7%)	0.000
Limb ischemia	13 (1.2%)	0 (0%)	1 (0.7%)	5 (1.8%)	2 (0.7%)	5 (2.4%)	0 (0%)	0 (0%)	0.396
Bowel ischemia	16 (1.5%)	0 (0%)	3 (2.1%)	3 (1.1%)	3 (1.0%)	2 (0.9%)	3 (2.5%)	2 (11.1%)	0.025
Surgical site infection	37 (3.4%)	2 (6.3%)	3 (2.1%)	10 (3.6%)	9 (3.1%)	11 (5.2%)	2 (1.7%)	0 (0%)	0.484
Acute renal failure	339 (31.0%)	10 (31.3%)	50 (34.2%)	89 (31.7%)	97 (33.9%)	56 (26.5%)	31 (26.3%)	6 (33.3%)	0.525
CRRT	127 (11.6%)	2 (6.3%)	14 (9.6%)	35 (12.5%)	28 (9.8%)	27 (12.8%)	16 (13.6%)	5 (27.8%)	0.246
Reexploration	64 (5.8%)	0 (0%)	6 (4.1%)	14 (5.0%)	26 (9.1%)	11 (5.2%)	6 (5.1%)	0 (0%)	0.119
ICU stay (days)	5 (8–3)	4 (6–3)	5 (7–3)	6 (10–3)	5 (8–3)	5 (9.5–3)	6 (10–4)	5 (12–3)	0.046
Hospital stay (days)	20.8 ± 13.1	21.9 ± 11.4	20.5 ± 10.0	21.7 ± 13.2	19.5 ± 11.5	21.8 ± 14.5	20.5 ± 17.2	14.4 ± 10.8	0.236

*ICH* intracranial hemorrhage, *GI* gastrointestinal, *CRRT* continuous renal replacement therapy, *ICU* intense care unit

### Follow-up

**Fig. 3:**
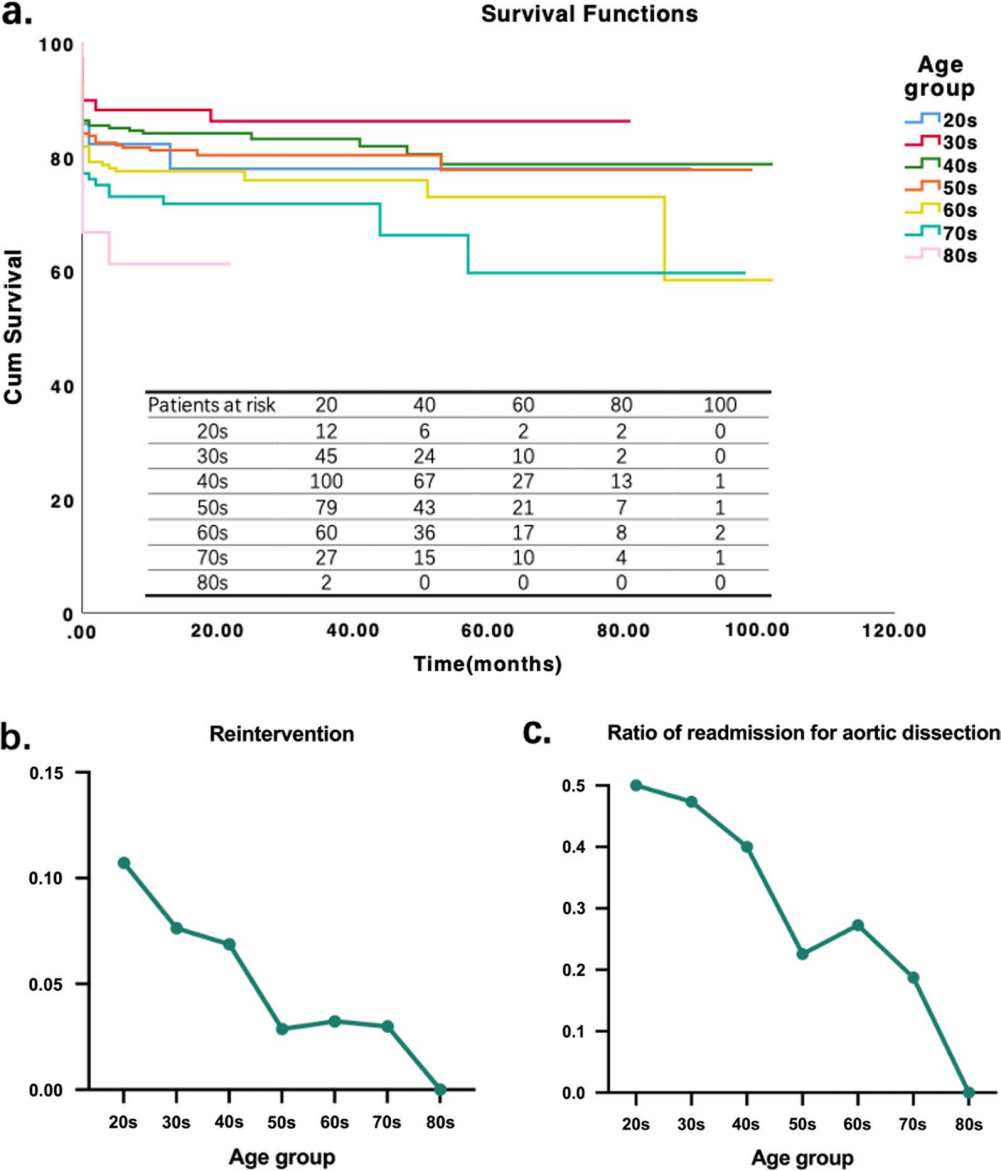
**a** Survival functions of different age groups. **b** The reintervention rate of different age groups. c. The ratio of readmission for aortic dissection

927 (84.9%) patients completed followed up and the median follow-up was 17 months (1–102 months). The 17-month survival rate was 82%. Thirty- eight discharged patients died during follow-up, 1 (3.1%) in 20 s group, 4 (12.5%) in 30 s group, 8 (25%) in 40 s group, 7 (21.9%) in 50 s group, 9 (28.1%) in 60 s group, 8 (25%) in 70 s group, and 1 (3.1%) in 80 s group. Figure 3a shows the mortality associated with age, the general tendency is that mortality increases with increasing age, 15.6% in 20 s group, 7.5% in 30 s group, 11.7% in 40 s group, 14.3% in 50 s group, 17.1% in 60 s group, 18.6% in 70 s group, and 33.3% in 80 s group; however, the mortality of patients over 70 years old decreased over these years while the ratio of these patients remains relatively stable. Among the patients who died, 10 (26.3%) from aortic rupture, 5 (13.2%) from neurological complications, and 2 (5.3%) patients died for stent leakage. 137 patients had readmissions, of whom 34 (24.8%) patients had thoracoabdominal aortic dissection/aneurysm which is the leading cause of readmission. Figure 3b, c shows the ratio of patients readmission for recurrence of aortic dissection, the tendency of readmission for abdominal aortic dissection decreased with age.

## Discussion

The average age of aTAAD patients was significantly younger in China, the results from Sino-RAD was 50.5 years [12] and 52 years in our center’s previous reports [5]^13^. In this study, we found the median age of patients of aTAAD is 52. Furthermore, there was a significant increasing number of aTAAD patients in 70 s and 80 s group who underwent surgical repair. The underlining reasons maybe multi-factorial. One of the main reasons is the increasing awareness of aTAAD among the public and emergency room physicians particularly since the introduction of our aTAAD refereral program (6 h life circle); the second is attributed to the improvement of surgical successful rate of aTAAD surgery; and 3rd maybe related to the recent increase of national and regional healthcare coverage [14] and especially in the second half of the study period (2016–2019). For older patients with aTAAD, the optimal treatment strategy is in debate depending on the risk and benefit ratio and the upper age limit is unknown. A study by Trimarchi et al. using IRAD data showed that patients older than 70 years old received higher rates of medical therapy than those of surgical repair (28.6% vs 10.9%; *P* < 0.0001), and there was no difference in survival between the two treatment strategies (55.8% vs 53.8%; *P* = 0.32) [1]. Our results are consistent with the above findings. The increasing risks and lower predictable late survival rate contributed to the lower proportion of open surgery. Many studies have shown that advanced age is related to poor postoperative survival, the long-term survival and the quality of daily life [15–17]. The poor outcome in patients of advanced age may have deterred the Septuagenarian and Octogenarian from undergoing extensive total arch surgery because there is no better alternative treatment such as endovascular repair.

Our present study also demonstrated that the proportion of patients who received surgical treatment over 70 years old remained relatively stable in last decade in our center. The higher 30 mortality in this group of patients suggested the negative impact of surgery on the postoperative recovery of patients with advanced age. In addition, the favorable long-term survival indicated that the simple or less extensive arch repair is the preferred surgery for patients over 70 years old. This finding is also corroborated by other studies [2]^18^.

Chest pain is the common clinical presentations of aTAAD for younger patients; however, the main etiology of aTAAD for patients in their 20 s is connective tissue disorders, such as Marfan syndrome while history of hypertension is more common in patients of aging 30, 40 and 50 s. Compared to the patients older than 70 years, the surgical strategies are totally different. More extensive surgical methods are applied for younger patients in order to avoid re-intervention because of aortic events [19–23]. Our study demonstrated that the recurrence rate of aTAAD was significantly lower in younger patients between 30 and 50 years old. The patients of 20 s group had the highest recurrent AD, consistant with the findings in Marfan’s syndrome paitents reported by Isselbacher et al. from the IRAD data [24]. The ratio of readmission for aortic dissection is also decreased with age. Because older patients had more complications than younger patients. These complications not only affect the time and strategies of surgery, but also affect the outcomes of the patients. And they had to have readmission to deal with the complications after surgery. On the contrary, the 20 s group had readmission most likely to deal with the recurrent aortic dissection. Therefore, extensive surgery strategy with higher surgical risk could not lower late recurrence and re-intervention.

The mode of the age was in the 40 s group, these patients were at the middle age of their life. It was necessary to pay more attention to their long term follow up and the quality of life. In the next years, we would focus on their changes and show what would happen to these post-operative aortic dissection patients. For the increasing number of hypertension patients in China, it was meaningful to know whether the 40 s group patients could totally recovery from the emergency surgery and go back to the society.

## Limitations

First, the retrospective study has its design limitation. Data were collected retrospectively so there are defects like incomplete, missing or inaccurate to report the event. The long-term survival rate would be underestimate as the follow-up interval is large and the follow-up time of some patients is shorter than one year. Second, the data obtained are of a single center and therefore could not represent the whole population. Third, as the number of patients in 20 s and 80 s group being limited, there is a need for further studies.

## Conclusions

Age is a major impact factor for aTAAD surgery. Old patients presented more comorbidities before surgery, the mortality and complications rate were significantly higher even with less invasive and conservative surgical therapy. But the favorable long-term survival indicated that the simple or less extensive arch repair is the preferred surgery for patients over 70 years old.

## Supplementary Information

**Additional file 1.** The number of patients admitted to Gulou hospital for aTAAD each year from 2011 to 2019.

## Data Availability

Data sharing is not applicable to this article as no datasets were generated or analysed during the current study.

## Abbreviations

aTAAD: Acute type A aortic dissection
AD: Aortic dissection
