# Supplementary figures and images for: The impact of age in acute type A aortic dissection: a retrospective study
Source: J Cardiothorac Surg. 2022 Mar 19;17:40. doi: 10.1186/s13019-022-01785-y (PMC8933992; doi:10.1186/s13019-022-01785-y)

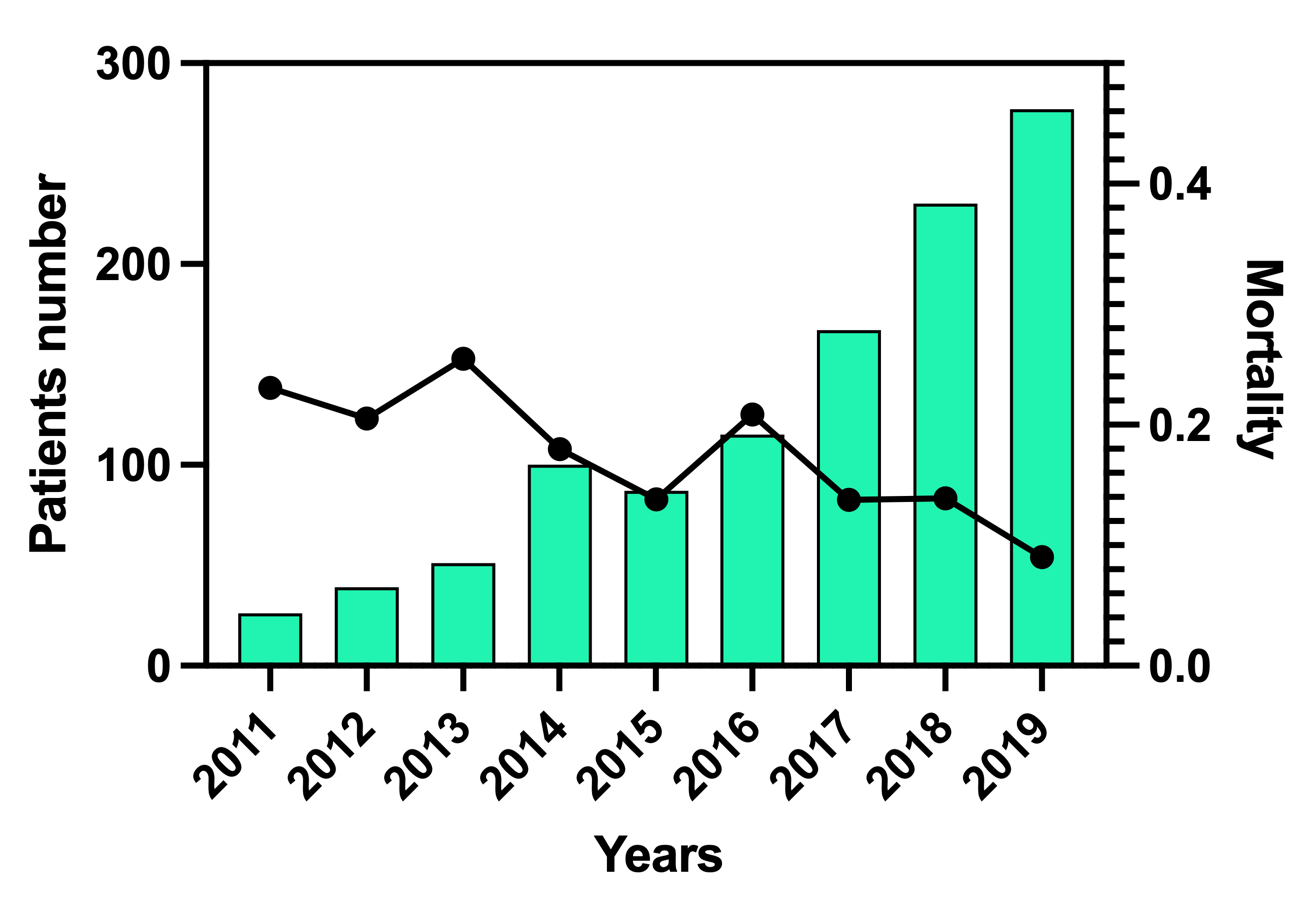

Supplement: Supplementary file 1 — Additional file 1. The number of patients admitted to Gulou hospital for aTAAD each year from 2011 to 2019. [file 13019_2022_1785_MOESM1_ESM.tiff]
